# Relationship between anterior mandibular bone thickness and the angulation of incisors and canines—a CBCT study

**DOI:** 10.1007/s00784-017-2255-3

**Published:** 2017-10-23

**Authors:** Agnieszka Srebrzyńska-Witek, Rafał Koszowski, Ingrid Różyło-Kalinowska

**Affiliations:** 1 Private Practice, Kossak-Szczuckiej Street 7/1, 40-578 Katowice, Poland; 2 Academic Center of Dentistry and Specialized Medicine, Pl. Akademicki 17, 41-902 Bytom, Poland; 3 Independent Unit of Propedeutics of Dentomaxillofacial Radiology, Medical University of Lublin, Karmelicka Street 7, 20-081 Lublin, Poland

**Keywords:** Cone-beam computed tomography, Imaging, three-dimensional, Mandible, Alveolar process

## Abstract

**Objectives:**

The morphology of the maxillary and mandibular alveolar cortex plays an important role in the planning of orthodontic treatment. Cone-beam computed tomography (CBCT) provides a precise demonstration of anatomical structures. Therefore, the aim of this paper was to evaluate what influence the position of incisors and canines have on the dimensions of the cortical and spongious bone of the anterior mandibular alveolar process.

**Materials and methods:**

The material consisted of 100 CBCT volumes (61 females and 39 males, aged 18–71 years) obtained by means of a Gendex GXCB-500 machine and analysed using i-CAT Vision and CorelDRAW 9 software. Several linear and angular measurements were taken of cortical and spongious mandibular, vestibular and lingual alveolar bone.

**Results:**

The thickness of the vestibular spongious bone increased around lateral incisors and canines together with dental axis inclination, as did the thickness of the lingual spongious bone around central incisors and canines with greater angles of vestibular cortex curvature. In all teeth, the thickness of lingual cancellous bone decreased along with increase of the angle of tooth inclination. In the case of almost all groups of teeth, the thickness of lingual cancellous bone around teeth declined as the angle of curvature of the cortical bone decreased. The rotation of mandibular incisors and canines did not affect the thickness of the surrounding bone.

**Conclusions:**

The position of teeth has little influence on vestibular bone thickness and is only significant around central incisors. In the case of almost all groups of teeth, the thickness of lingual spongious bone around teeth declined as the angle of curvature of the cortical bone decreased.

**Clinical relevance:**

CBCT is a diagnostic tool that provides detailed information on the dimensions of the anterior dentate mandibular alveolar process.

## Introduction

Due to its specific anatomy, the anterior mandible is an area that poses considerable diagnostic and therapeutic problems. This is due to the relatively small dimensions of teeth as well as the small distances between them, and these difficulties may be further intensified by frequent dental crowding [1–3]. The vestibular cortical bone of the mandibular alveolar process is of utmost importance as its dimensions influence the aesthetics of the patient’s smile. This structure is prone to resorption, e.g. during the course of periodontal bone disease as well as during orthodontic or implantological treatment. Moreover, the profile of the periodontal bone, mainly the vestibular cortical bone, affects the healing of post-extraction wounds. Bone remodelling occurs after any dental extraction that leads to atrophy, mainly in the transsectal plane, and which is more advanced on the vestibular side of the jaw. This hampers or even makes it impossible to manufacture fixed prosthetic appliances, either conventional devices or those based on dental implants [4, 5].

Provided the anatomy of the recipient site is thoroughly assessed by the dentist, the latter is able to choose a suitable implant with the desired shape and dimensions, plan its final position and decide whether further augmentation is necessary. Correct preoperative diagnostics also make it possible to predict potential bone resorption. Immediate implant placement combined with simultaneous bone augmentation is becoming increasingly common. The status and thickness of the vestibular mandibular cortex is of key importance when choosing the correct treatment options [6].

The morphology of the maxillary and mandibular alveolar cortex plays an important role in the planning of orthodontic treatment, especially in cases where there is a considerable discrepancy between the volume of teeth and the amount of space available in the dental arches. The movement and inclination of teeth towards the oral vestibule often results in reduced thickness of the external cortex or in its discontinuity in the form of fenestrations and/or dehiscences. Orthodontic forces applied during this kind of treatment increase tissue strain and result in reduced keratinized gingiva thickness. As a result, it may become too thin for the progenitor cells responsible for bone formation. Gingival recessions may develop, and this complication is more common around mandibular incisors [7].

When the maxillary and mandibular alveolar cortex is thin, periodontal surgery is recommended so as to increase its thickness before embarking on any orthodontic expansion of the dental arch. Such surgery is based on transplanting the hard palate mucosa or subepithelial connective tissue [8–10].

The recent development of radiological imaging in the form of cone-beam computed tomography (CBCT) provides a more precise demonstration of anatomical structures and the detection of pathological lesions. CBCT has proved extremely useful in dentistry due to its relatively low exposure dose (when compared with medical CT) and high resolution [6]. CBCT scanning is frequently used in the planning of implantological and orthodontic treatment. We thus assumed that application of CBCT may supply crucial information on the relationships between the morphology of the dentate anterior mandible and the position of teeth. Therefore, the aim of this paper is to evaluate what influence the position of inferior incisors and canines have on the dimensions of the cortical and spongious bone of the anterior mandibular alveolar process.

## Material and methods

The material consisted of cone-beam computed tomography volumes obtained from the Radiological Lab of the Jomadent Health Center in Dąbrowa Górnicza (Poland) from 2010 to 2012. The study was approved by the local bioethical committee (KNW/0022/KB/190/13). All the CBCT examinations were performed due to clinical indications and not for the purpose of this study. The selection criteria for patients were as follows: age over 18 years and all upper and lower incisors, canines, premolars and at least the first molars present in the dental arches. The exclusion criteria were as follows: orthodontic treatment (current or previous), prosthetic crowns on mandibular incisors and canines, the presence of any lesion (e.g. a tumour, cyst, periapical lesion, supernumerary tooth), foreign bodies in the anterior mandible, previous surgery on the anterior mandible as well as CBCT volumes of inferior quality (artefacts, incomplete coverage of the anterior mandible, patient movement, incorrect exposure settings, low resolution) and medication intake affecting bone metabolism (such as bisphosfonates, calcium).

Eventually, 100 CBCT volumes from 61 females and 39 males aged from 18 to 71 years (mean age 41.34 years, 43.95 years in males and 39.67 years in females) qualified for the retrospective analysis. Statistical analysis was performed in two age groups—between 18 and 49 years of age (70 CBCT volumes taken in 45 women and 25 men) and between 50 and 71 years of age (the remaining 30 volumes including 16 women and 14 men). All the CBCTs were obtained with a Gendex GXCB-500 machine, and the following exposure parameters were applied: 120 kV, 5 mA, exposure time between 6 and 8 s and voxel size 0.3 mm. The region of interest included upper and lower dental arches within a cylindrical field of view of 8 × 8 cm. The slices obtained in this way were analysed using specially designed i-CAT Vision software, which was unable to perform all the planned linear and angular measurements. Therefore, the authors developed their own method so as to transfer selected slices from i-CAT Vision software to CorelDRAW 9 software (serial number DX9XR—6840J50620) by means of IrfanView software (by Irfan Skiljan).

**Fig.1:**
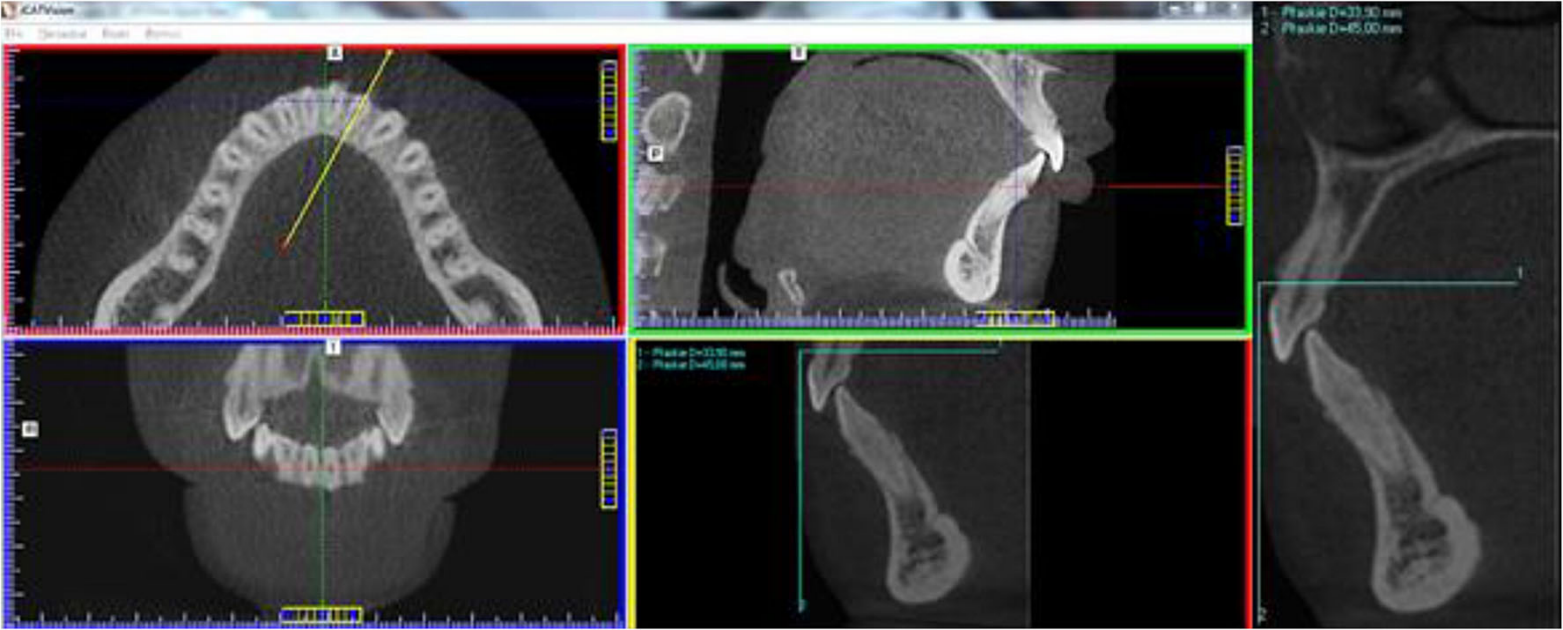
A drawing of the line determining the plane of a cross-sectional slice in the area of the examined tooth as well as a drawing of the calibration lines

Image analysis consisted of measurements taken in the mandible in the area of teeth 43, 42, 41, 31, 32 and 33. In the first step, an axial slice at the cervix level of the mandibular anterior teeth was formed with i-CAT Vision software. Then, lines were drawn at each tooth and these lines crossed at two points: the first was located at the maximum convexity of the vestibular outline of the tooth and the second similarly at maximum lingual tooth convexity. The line was always drawn in the middle of the cross section of the root canal (Fig. 1). These cross-sectional images were then exported to CorelDRAW 9. To maintain measurement accuracy, two calibrating lines of known length, perpendicular one to another, were drawn using IrfanView software (Fig. 1). Before proceeding with further measurements in CorelDRAW 9, the correct size of the exported image was set using the abovementioned calibration lines so as to ensure a highly accurate linear and angular measurement.

The following parameters were measured on cross-sectional slices of six anterior mandibular teeth:

1. The thickness of the vestibular and lingual cortex at four levels
2. The thickness of the vestibular and lingual spongious bone at four levels
3. The angulation of the cortical bone
4. The angulation of long axes of teeth in relationship to the mandibular base
5. The angulation of rotation of teeth in relationship to the midline

The secondary points and lines were determined for measurements of cortical and spongious bone thickness, such as the tooth axis running through the incisal edge or cusp and root apex. The image was then rotated so that the dental axis was parallel to the Y axis. Next, a line was established perpendicular to the dental axis passing through the cemento-enamel junction (CEJ), and then four lines were drawn:

1. Halfway between the CEJ and the radiological tooth apex
2. At 6 mm above the root apex perpendicular to the tooth axis running 6 mm above the radiological root apex
3. At 3 mm above the root apex
4. At the root apex

**Fig. 2:**
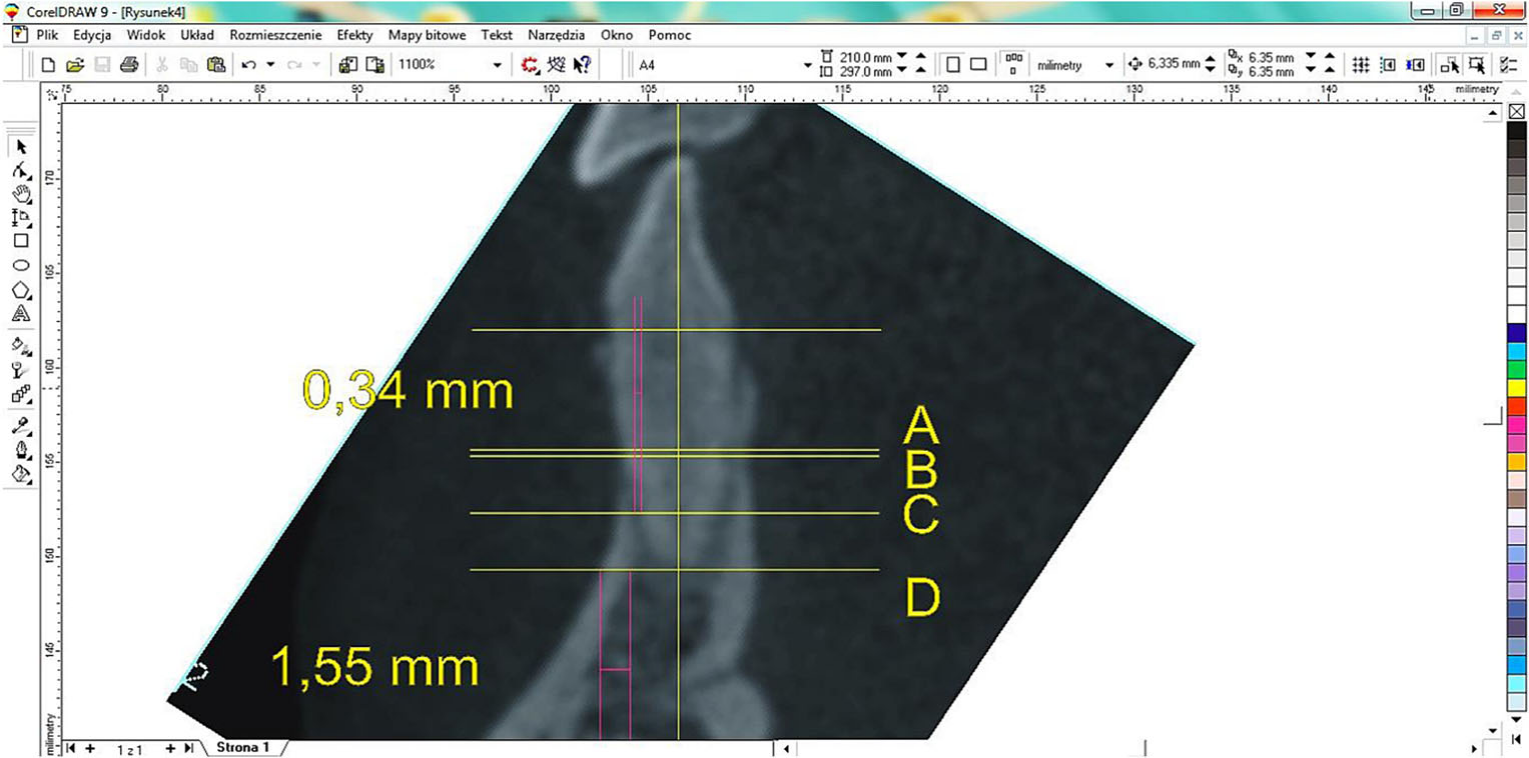
Measurements of the thickness of cortical and spongious bones along determined accessory lines

These lines determined the areas where measurements were taken of the vestibular and lingual cortex as well as the spongious bone (Fig. 2).

**Fig. 3:**
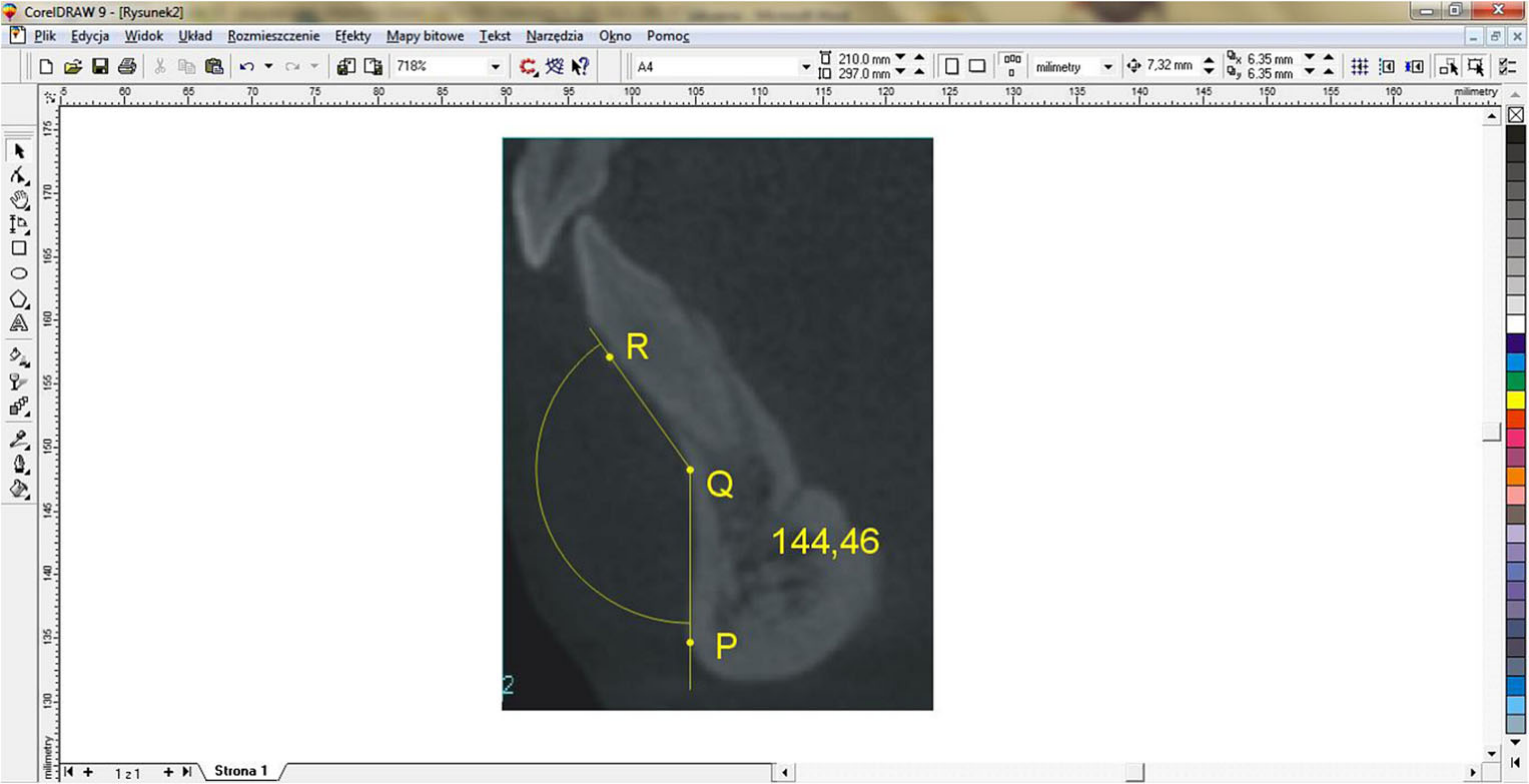
Measurements of the curvature of the vestibular alveolar bone of the mandible

To evaluate the curvature of the vestibular alveolar bone and the mandibular body, the following points were determined:

- Point Q, located at the deepest (most lingual) point on the curvature of the vestibular cortex
- Point P, located at the most labial point on the cortical bone of the mandibular body
- Point R, located at the top of the vestibular cortical bone (Fig. 3)

**Fig. 4:**
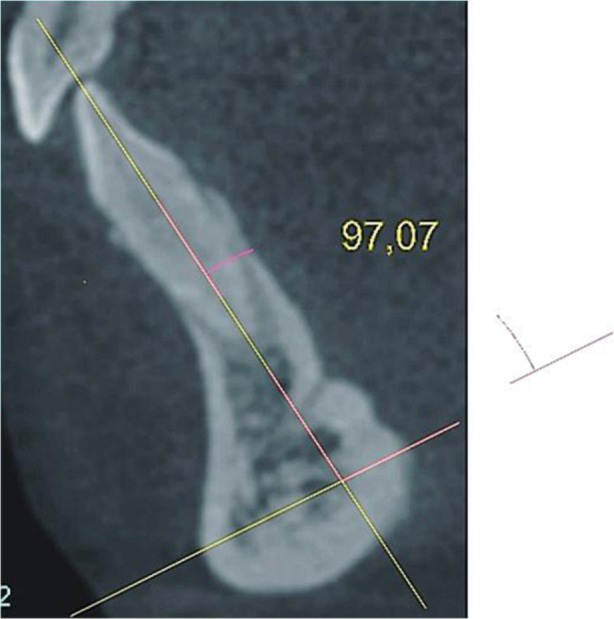
Measurements of the angulation of a tooth in relationship to the mandibular body line

We also estimated the angulation of each examined tooth in relationship to the mandibular bony on the lingual side between two lines. The first line intersected the points located at the most anterior and posterior points on the inferior margin of the cross-sectional slice of the mandibular body, while the second line connected the incisal edge and apex of the given tooth (Fig. 4).

**Fig. 5:**
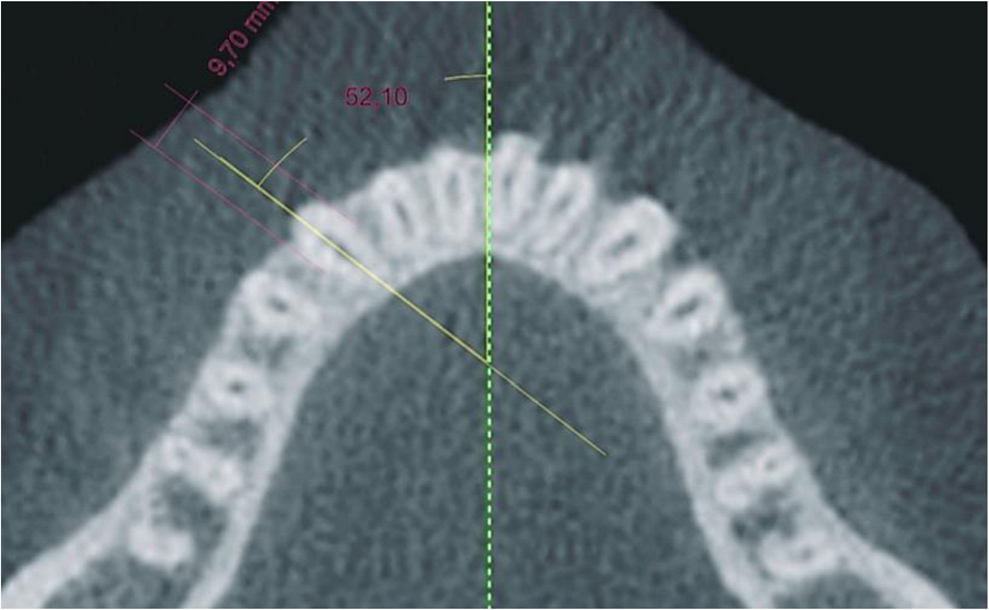
Measurements of tooth rotation in an axial slice

Tooth rotation in relation to the midline was estimated on calibrated axial slices using CorelDRAW 9 (Fig. 5).

Every linear and angular measurement was taken three times on three consecutive days by the same observer (ASW), and the mean value was calculated.

The statistical analysis was performed using Statistica for Windows software version 10 (demo version). Apart from descriptive statistics methods, Student’s *t* test, ANOVA and Pearson’s correlation coefficient were also used. The significance level was *α* = 0.05.

## Results

**Fig. 6:**
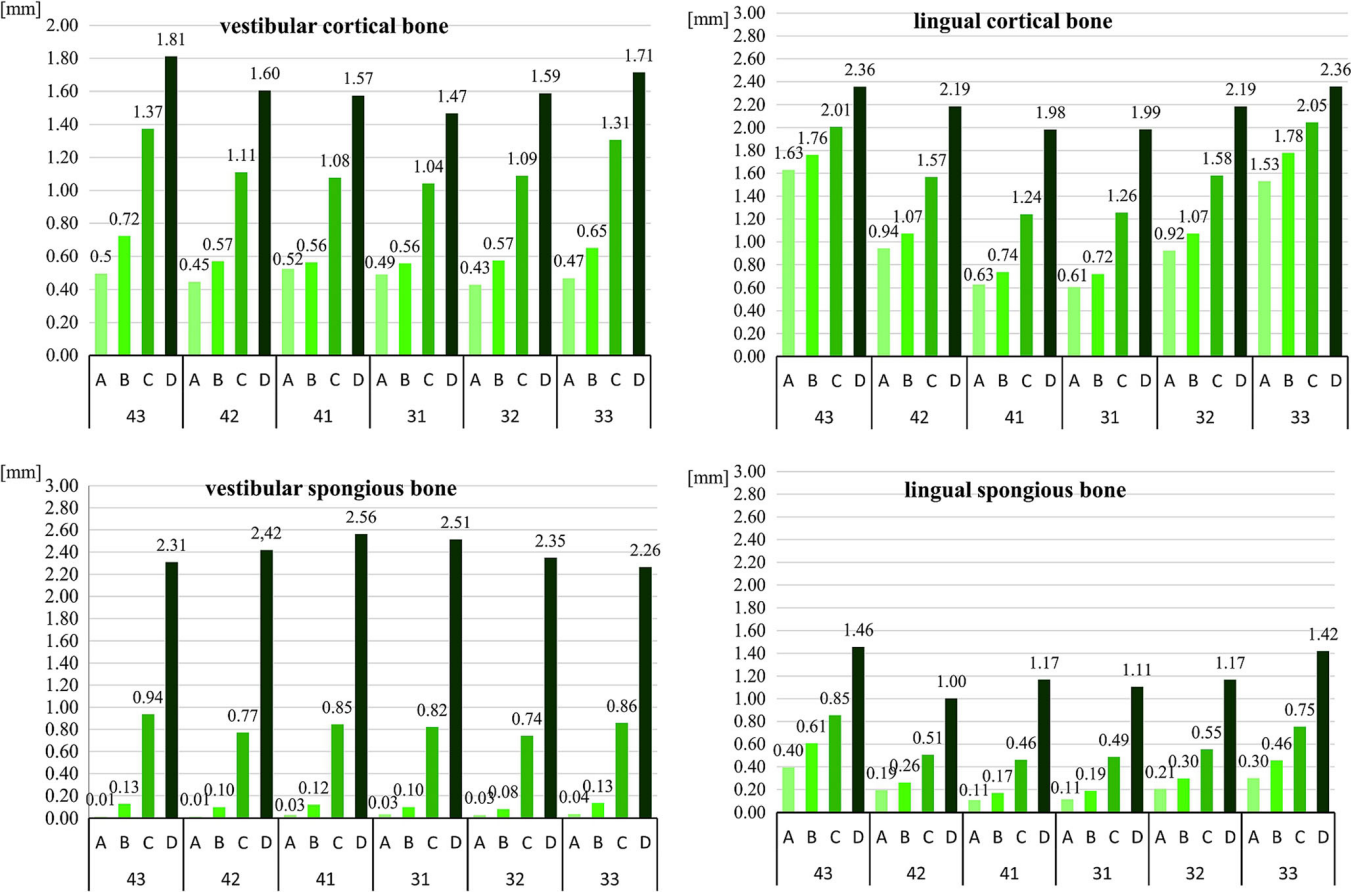
The mean thickness of vestibular as well as lingual cortical and spongious bones in the region of mandibular incisors and canines according to the distance from the root apex

The mean thickness of the vestibular cortex was 0.97 mm ± 0.24 mm. The vestibular spongious bone measured on average 0.84 mm ± 0.49 mm. The cortical and spongious bone was least thick in the middle of roots, but increased in thickness which increased towards the apices. The lowest values at this level were found for the vestibular spongious bone, which was sometimes non-existent (Fig. 6).

The mean thickness of the lingual alveolar cortex was 1.51 mm ± 0.35 mm, while the mean thickness of the spongious bone was 0.59 mm ± 0.31 mm. Again, the cortical and spongious bone was thinnest in the middle of the roots and increased in thickness towards the apices (Fig. 6). No statistically significant differences were observed between males and females with regard to the thickness of the vestibular spongious bone or the lingual cortical and spongious bone.

The mean angulation of the vestibular cortex was 142.74° ± 7.00°, and there was a statistically significant difference between this angle around lateral incisors and canines (*p* = 0.021). The angulation of the long axis of a tooth in relation to the mandibular body equalled on average 94.29° ± 9.30°. The values of this angle were statistically lower around canines than around lateral incisors (*p* = 0.029) (Table 1). However, no differences between genders were found.

**Table 1:** Mean values of examined angles regarding type of tooth

Angle	Angle of curvature of vestibular alveolar bone (*p* = 0.021*)	Angle of inclination of long axis of tooth in relationship to mandibular body line (*p* = 0.029 **)	Angle of tooth rotation
Type of tooth			
Central incisor	142.15° ± 8.23°	96.40° ± 12.92° **	6.91° ± 3.58°
Lateral incisor	141.72° ± 7.03° *	93.65° ± 10.65°	18.39° ± 6.37°
Canine	144.36° ± 8.93° *	92.82° ± 9.85° **	43.29° ± 9.86°

Single asterisks in the 2nd column and double asterisks in the 3rd column indicate the pair of results (out of 3) that are different and they are statistically significant with p value showed in the 1st line

**Fig. 7:**
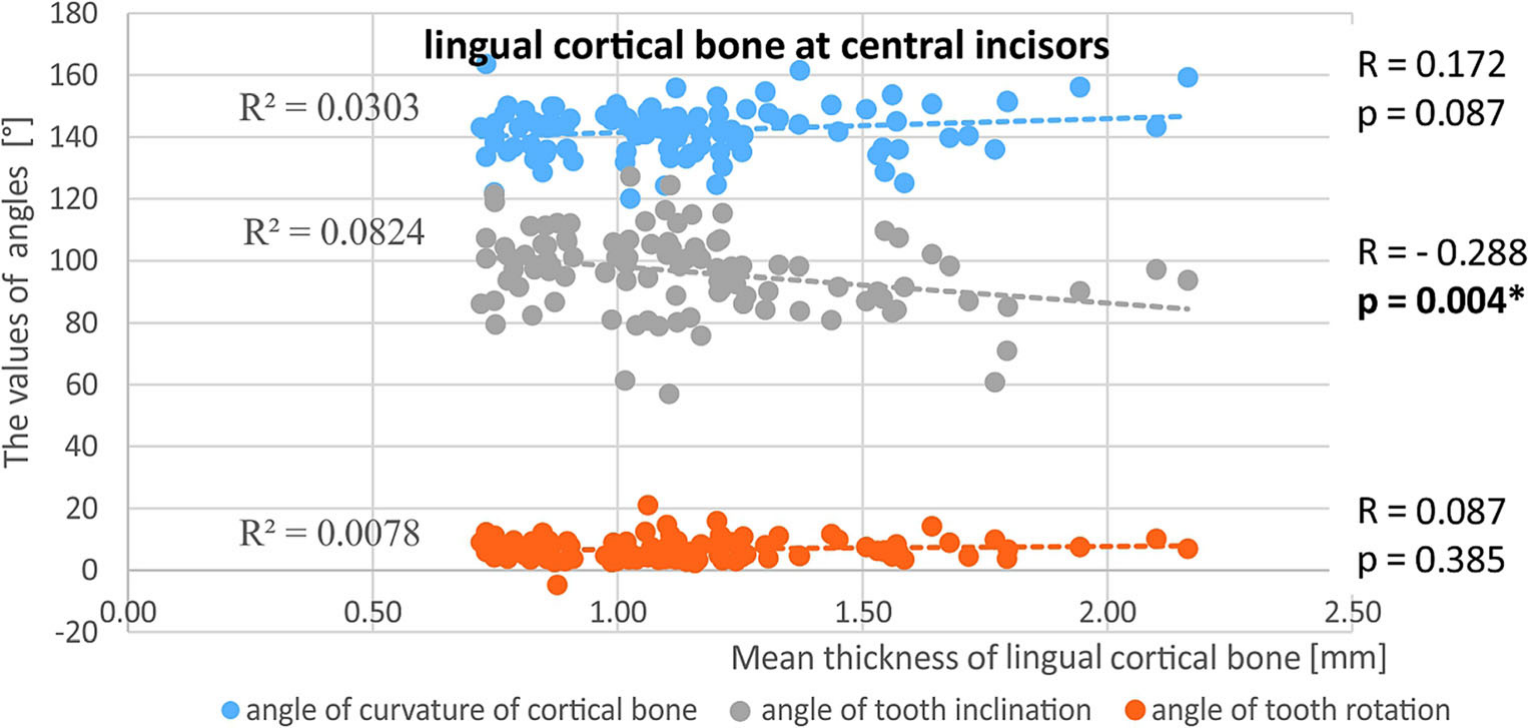
The relationship between the mean width of the lingual cortical bone around the central incisors and the angle of curvature of the cortical bone, the angle of tooth inclination and the angle of tooth rotation

No relationship was observed between cortical bone thickness (on either the vestibular or lingual side) and the angulation of the long axis of the tooth towards the mandibular body, the angulation of the cortical plate and tooth rotation. The only exception was the central incisors, where a decrease in tooth angulation in relation to the mandibular body correlated with an increase in lingual cortex thickness (Fig. 7).

**Fig. 8:**
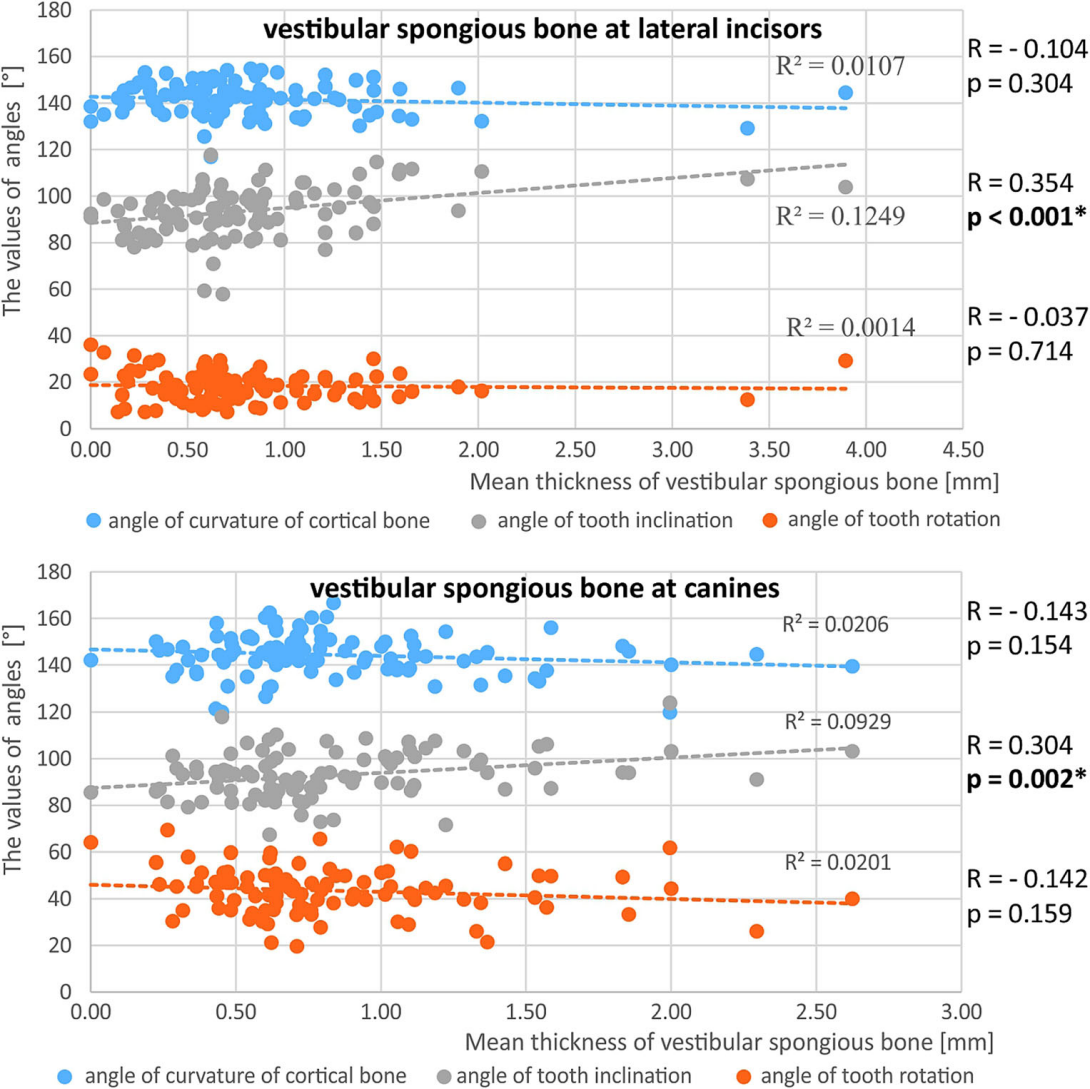
The relationship between the mean width of the vestibular spongious bone around the lateral incisors and canines and the angle of curvature of the cortical bone, the angle of tooth inclination and the angle of tooth rotation

**Fig. 9:**
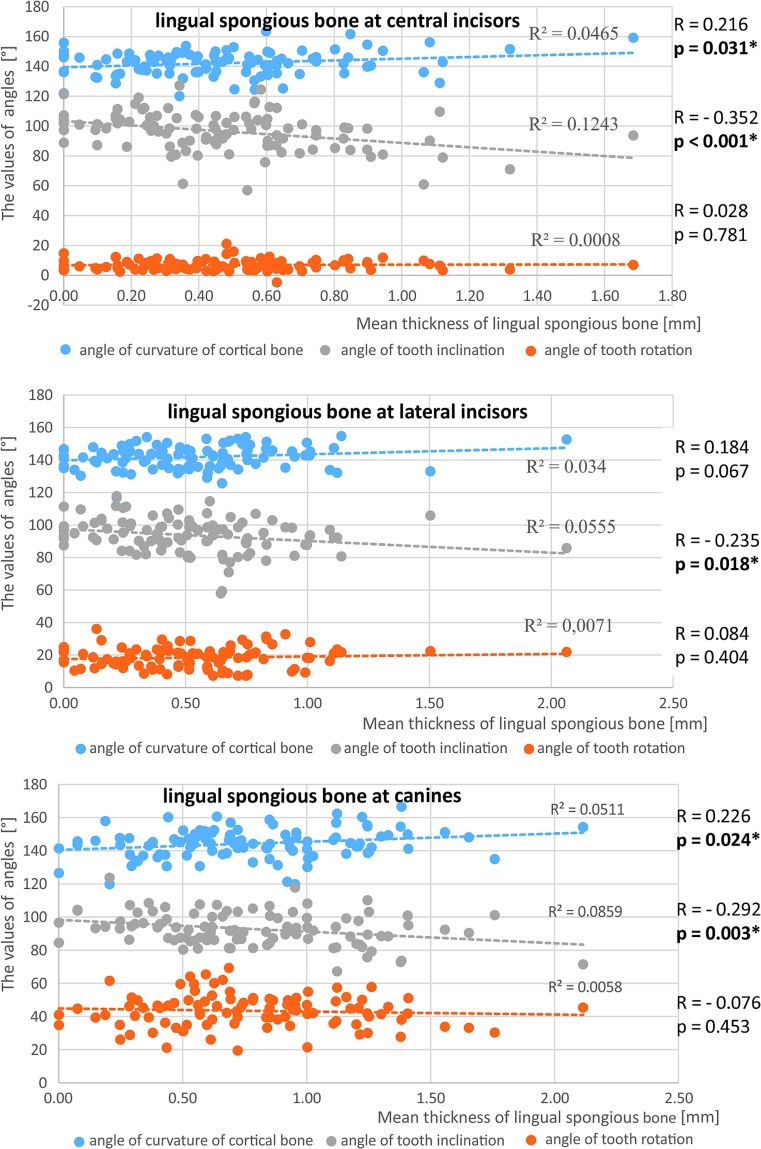
The relationship between the mean width of the lingual spongious bone around the incisors and the canines and the angle of curvature of the cortical bone, the angle of tooth inclination and the angle of tooth rotation

Spongious bone thickness was greater around the lateral incisors and canines when the angulation of the tooth axis increased (Fig. 8). A positive correlation existed between lingual spongious bone thickness in all dental groups on the one hand and increasing angulation of teeth in relation to mandibular line on the other. The greater the angulation of the buccal cortex, the greater the thickness of the lingual spongious bone (Fig. 9).

**Fig. 10:**
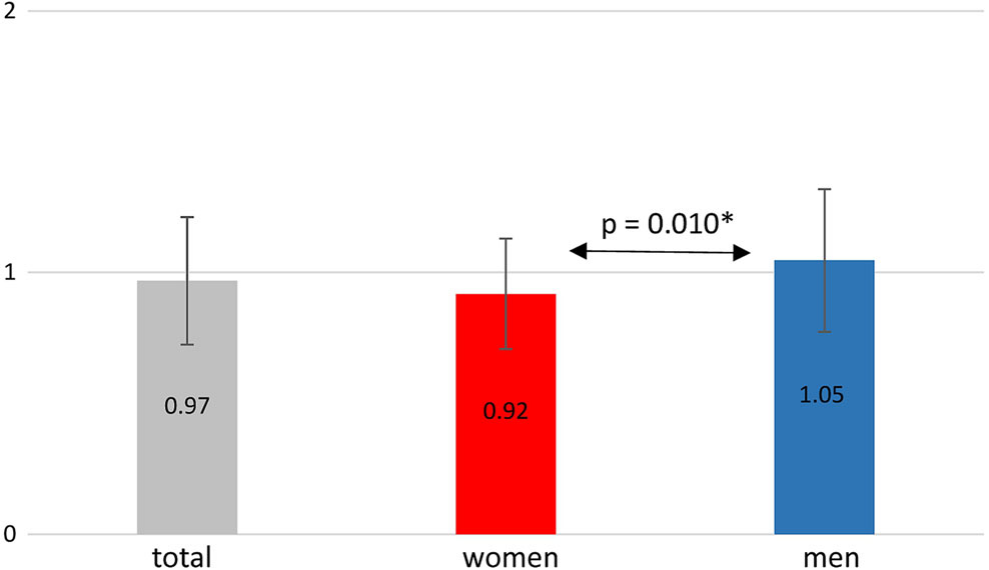
The mean thickness of the vestibular cortex, according to gender

Regarding the influence of gender on the measured parameters, the mean thickness of the buccal cortex was bigger in males than in females, and this difference was statistically significant (*p* = 0.01) (Fig. 10). There were no statistically significant relationships between females and males regarding the width of the lingual cortex as well as the buccal and lingual cancellous bone.

When the age of the patients was taken under account, there were no significant differences in mean buccal cortex width below and over 50 years of age, both in males and females (Table 2). Lingual cortex thickness was significantly higher in females aged over 50 years (*p* < 0.05) (Table 3). There was no such relationship in age groups of males (Table 3). Age did not influence the width of the buccal cancellous bone in females and males (Table 4), while the lingual cancellous bone was significantly thicker in females aged over 50 years (*p* = 0.008) than in the younger subjects (Table 5). Again, no such relationships were determined in the males (Table 5). The values of angulation of the vestibular cortex and of tooth angulation in relation to the base of the mandible did not significantly differ neither in males and females nor in age groups (Tables 6 and 7).

**Table 2:** Mean thickness of the buccal cortex taking under account gender and age groups

	Males and females			Females			Males		
	Total	< 50	≥ 50	Total	< 50	≥ 50	Total	< 50	≥ 50
Mean	0.97	0.96	0.99	0.92	0.91	0.94	1.05	1.05	1.05
Standard error	0.02	0.03	0.05	0.03	0.03	0.06	0.04	0.05	0.08
Standard deviation	0.24	0.23	0.27	0.21	0.20	0.24	0.27	0.26	0.30
Confidence interval (95.0%)	0.05	0.06	0.10	0.05	0.06	0.13	0.09	0.10	0.16
		*p* = 0.572			*p* = 0.646			*p* = 0.995

**Table 3:** Mean thickness of the lingual cortex taking under account gender and age groups

	Males and females			Females			Males		
	Total	< 50	≥ 50	Total	< 50	≥ 50	Total	< 50	≥ 50
Mean	1.51	1.48	1.56	1.55	1.50	1.68	1.45	1.45	1.43
Standard error	0.03	0.04	0.06	0.04	0.05	0.07	0.06	0.09	0.09
Standard deviation	0.35	0.36	0.33	0.31	0.32	0.27	0.40	0.42	0.35
Confidence interval (95.0%)	0.07	0.09	0.12	0.08	0.10	0.14	0.13	0.18	0.20
		*p* = 0.283			*p* = 0.048*			*p* = 0.887

**Table 4:** Mean thickness of buccal cancellous bone taking under account gender and age groups

	Males and females			Females			Males		
	Total	< 50	≥ 50	Total	< 50	≥ 50	Total	< 50	≥ 50
Mean	0.84	0.83	0.87	0.78	0.79	0.74	0.95	0.91	1.01
Standard error	0.05	0.05	0.10	0.05	0.06	0.09	0.10	0.12	0.19
Standard deviation	0.49	0.46	0.55	0.36	0.37	0.34	0.63	0.59	0.71
Confidence interval (95.0%)	0.10	0.11	0.21	0.09	0.11	0.18	0.20	0.24	0.41
		*p* = 0.712			*p* = 0.626			*p* = 0.690

**Table 5:** Mean thickness of lingual cancellous bone taking under account gender and age groups

	Males and females			Females			Males		
	Total	< 50	≥ 50	Total	< 50	≥ 50	Total	< 50	≥ 50
Mean	0.59	0.53	0.73	0.57	0.52	0.73	0.63	0.55	0.75
Standard error	0.03	0.04	0.05	0.03	0.04	0.05	0.06	0.08	0.08
Standard deviation	0.31	0.32	0.26	0.27	0.27	0.22	0.37	0.39	0.30
Confidence interval (95.0%)	0.06	0.08	0.10	0.07	0.08	0.12	0.12	0.16	0.17
		*p* = 0.002*			*p* = 0.008*			*p* = 0.102	

* indicates statistically significant differences

**Table 6:** Mean angulation of the buccal cortex taking under account gender and age groups

	Males and females			Females			Males		
	Total	< 50	≥ 50	Total	< 50	≥ 50	Total	< 50	≥ 50
Mean	142.74	142.44	143.45	143.52	143.72	142.97	141.52	140.13	144.01
Standard error	0.66	0.80	1.18	0.74	0.87	1.44	1.22	1.52	1.96
Standard deviation	7.00	6.68	6.46	5.77	5.82	5.76	7.64	7.59	7.35
Confidence interval (95.0%)	1.31	1.59	2.41	1.48	1.75	3.07	2.48	3.13	4.24
		*p* = 0.483			*p* = 0.657			*p* = 0.130

**Table 7:** Mean angulation of teeth regarding the base of the mandible taking under account gender and age groups

	Males and females			Females			Males		
	Total	< 50	≥ 50	Total	< 50	≥ 50	Total	< 50	≥ 50
Mean	94.29	95.78	91.70	94.69	95.42	92.63	94.36	96.44	90.64
Standard error	0.93	1.23	1.81	1.08	1.21	2.30	2.06	2.72	2.94
Standard deviation	9.30	10.32	9.94	8.41	8.10	9.18	12.89	13.60	10.99
Confidence interval (95.0%)	1.85	2.50	3.71	2.15	2.43	4.89	4.18	5.61	6.34
		*p* = 0.070			*p* = 0.259			*p* = 0.181	

## Discussion

One of the advantages of CBCT is the absence of any image distortion or image magnification. The mean error of linear measurement is 0.1–0.20 mm, while panoramic image distortion may reach 20%. It should be emphasised that the precision of linear measurements is highest in the middle of the volume and increases towards the edges of the field of view [11–15]. The sensitivity and specificity of CBCT in detecting fenestrations was estimated at 90% and in the case of dehiscence specificity reached 95%, while sensitivity was only 40% [16, 17].

Many studies have compared linear measurements in both CBCT and the real dimensions of skulls. The conclusion to be drawn from them is that the method was reliable, but measurement precision was limited by voxel size. According to Kobayashi et al. [18], precision is 0.22 mm ± 0.5 mm when the voxel size is 0.125 mm, while Mischkowski et al. [19] report a figure of 0.26 mm ± 0.18 mm. Timock et al. [20] estimated precision at 0.30 mm ± 0.27 mm, and Leung et al. [16] at 0.6 mm ± 0.8 mm when the voxel size was 0.38 mm. The voxel size in our own study was 0.3 mm.

Correctly determining reference points is crucial for ensuring measurement precision, and it is easiest to use the interfaces of structures characterised by different densities e.g. enamel and cementum. In such cases, determining the reference point with precision depends on the voxel size. When a reference point is located on tissue with a similar density to that on the alveolar ridge, it is much more difficult to select the right area.

Leung et al. [16] determined the accuracy of determining the cemento-enamel junction at 0.4 mm ± 0.3 mm, and the vestibular cortex ridge at 0.6 mm ± 0.8 mm. This difference is due to the limitations imposed by the spatial image resolution, defined as the smallest distance allowing for separate imaging of two parallel lines or two points. When accuracy equals 0.6 mm, all bony areas thinner than this will be visualised as areas with no bone at all, and it is the minimum bone thickness that is measurable. In practice, this leads to errors in image interpretation and to the overdiagnosis of missing bone when it is actually present, but thinner than spatial image resolution [12, 16]. The authors took this into account when determining cortical and spongious bone thickness, while the alveolar lamina dura was included in measurements of spongious bone or cortical bone when the spongiosa was too narrow to be measured on its own.

The available literature mostly deals with CBCT analyses of the maxillary vestibular cortex. On the other hand, few papers have focused on the morphology of the mandibular vestibular bone. To the best of our knowledge, there are few publications reporting on the thickness of both vestibular and lingual mandibular bone that also provide an analysis of the position of anterior teeth.

The authors’ own results were similar to those obtained by Zekry et al. [21]. The latter demonstrated that mean cortical bone thickness at 3 mm below the alveolar ridge was 0.89 ± 0.3 mm and increased from the incisors towards distal teeth. Baysal et al. [22] analysed the vestibular and lingual cortices at the apices of the central incisors in CBCT and found that the vestibular cortex measured between 1.41 mm ± 0.45 mm and 1.98 mm ± 0.46 mm depending on the type of malocclusion, while on the lingual side it ranged from 1.79 mm ± 0.45 mm up to 2.17 ± 0.44 mm.

Swasty et al. [23] estimated cortical bone thickness on the vestibular and lingual sides of interdental spaces in the mandible on the basis of CBCT. They observed that the buccal cortex was thinner around anterior teeth and equalled 1.8 mm, while its thickness increased towards the distal sides to reach 3.2 mm. These values are higher than those obtained in our own study because the quoted authors used different reference points located at interdental spaces containing more bone tissue than the vestibular and lingual surfaces of teeth.

Rossell et al. [5] elaborated their own method for measuring the vestibular cortex around inferior central incisors on the basis of two-dimensional X-rays. They determined that the mean cortical thickness at 3 mm below the alveolar ridge equalled 0.66 mm ± 0.27 mm [5]. In our own study, such thickness in the middle of the root length was 0.34 mm ± 0.34 mm for central incisors.

A CBCT study by Lee et al. [24] focused on the morphology of the maxillary alveolar processes around incisors and canines. They discovered that the mean thickness of the vestibular cortex in the middle of root length was 2.36 mm ± 0.6 mm for central incisors, 1.83 mm ± 0.96 mm for lateral incisors and 2.95 mm ± 1.63 mm for canines [24]. These values differ significantly from the authors’ own results for the anterior mandible, where no dimensions exceeded 0.5 mm. According to other authors, the thickness of the maxillary cortex at one half of the root length falls between 0.5 and 1.05 mm. Hence, again these values are higher than in the present study and other papers dealing with mandibular morphology [25–30].

This tendency has been confirmed using other methods. Ghassemian et al. [4] analysed 66 spiral computed tomography studies on the thickness of the anterior maxillary cortex at 3 mm from the alveolar ridge. The thickness determined by this method ranged from 1.41 to 1.73 mm, depending on the examined tooth. Huynh-Ba et al. [31] examined 93 patients qualifying for tooth extraction and immediate implant placement in the maxilla. They measured the thickness of the buccal and lingual bone at 1 mm from the alveolar ridge in vivo directly after tooth extraction. The mean buccal thickness at this level equalled 1 and 1.2 mm on the lingual side.

Our own study showed that the lingual cortex was thicker than the vestibular cortex. Moreover, it was wider around the canines than the incisors. As far as spongious bone is concerned, it is very thin or even non-existent in the middle of the root length on the vestibular side of the mandible, and in this study, such a situation was observed in 94.3% of cases. Therefore, in many papers the entire bone covering the vestibular root surface is called cortical and the rudimentary spongious bone is neglected. According to our own results, the thickness of the vestibular spongious bone increases apically and is similar around canines and incisors. On the lingual side, its dimensions also increase towards the root apex but they are largest around canines and smallest around the central incisors. Gracco et al. [32] investigated the width of the entire alveolar ridge at the incisal region of the mandible in various facial types by means of CBCT as well as the width of the spongious bone at the apex of the roots depending on the vertical pattern of facial growth. They concluded that at the vestibular side the thickness ranged between 2.33 and 3.73 mm, while that at the lingual side is 1.14–1.98 mm. These values were close to our own results—2.38–2.53 mm at the buccal side and 1.09–1.10 mm at the lingual side of lower incisors. As for the most part, the total thickness of the cortical and spongious bone does not exceed 1 mm and does not reach the recommended 2 mm; treatment planning should include more lingual implant placement than the original tooth position. The cortical bone is wider on this side, and more bone is left to cover the implant from the buccal side.

It seems that age and gender may influence the quantity of bone surrounding anterior teeth. It was confirmed by Januário et al. [25], Nowzari et al. [30] and Wang et al. [27] who studied anterior maxillary morphology, while Ozdemir et al. [33] took both the maxilla and mandible under consideration. They all noted lower bone thickness in females compared with males and a tendency for thickness to decrease with age. On the other hand, while Braut et al. [28] did not register any gender differences they did note that cortical bone thickness decreased with age. In the authors’ own study, it was proved that only the buccal cortex was significantly thicker in males than in females. However, there were statistically significant higher values of lingual cortex and spongious bone in females aged over 50 years in comparison with younger females. Jonasson et al. [34, 35] carried out a 5-year prospective study on perimenopausal women and observed that the bucco-lingual dimension of the alveolar process of the dentate mandible decreased with age, mainly in the lateral areas. On the other hand, Swasty et al. [23] did not observe any difference between the genders regarding the thickness of the cortex of the whole mandible, but they found an increase in thickness with age with peak width in the age group 40–49 years.

The morphology of the anterior mandible is also influenced by the facial patterns of individual patients, especially when disproportions between anterior and posterior height are observed because the mandibular bone is affected by muscle attachments. It is believed that patients with hyperdivergence, a long face, have thinner bone both on the buccal and the oral sides compared with individuals with a short face type (hypodivergence) [17, 36, 37]. This was confirmed by Gracco et al. as well. [32]. Moreover, Baysal et al. [22] analysed a group of patients with class I and II malocclusions and came to the conclusion that the vestibular cortex was thinner in class II patients than in their class I counterparts while individuals with a high SN-GoGn angle were characterised by a narrower cortex than class II patients with an average SN-GoGn angle. In our own study, we did not investigate malocclusions.

In orthodontics, the angulation of anterior teeth is estimated using lateral cephalometric radiographs and cephalometric analysis. The Schwarz system evaluates the angle formed by a cross section of the long axis of a tooth with the mandibular base plane (MP) determined by the Gn point and the point located in the antegonial notch. In adults, this angle should measure 90° ± 5° [38]. Steiner, using an approach modified by Kaminek, assessed the LI/ML angle running between the long axis of the lower incisor LI and the ML line which is tangential to the inferior mandibular margin. The LI/ML equals on average 94° ± 7° [39]. Such measurements can be carried out in a large FoV CBCT with specially designed cephalometric software. However, an analysis of three-dimensional reconstructions may involve considerable measurement errors [40, 41]. In our own study, FoV was not large enough to allow us to determine the ML and MP lines and we did not use a cephalostat. Therefore, dental angulation was estimated using our own modified approach based on cross-sectional slices called the mandibular base line. The angulation was similar to the reference values found in cephalometric analysis systems and statistically significantly higher for canines than for incisors. It should be pointed out that values derived from cephalometric analyses concern only inferior central incisors, which according to our own study are more inclined than canines [38, 39]. On the other hand, it is important to note that the genial tuberosity located in the region of the inferior incisors may influence the measurement results as the line of the mandibular body is determined on the basis of the most anterior point. If the tuberosity is large, it may affect the values of this angle. In our own study, some correlations were found between bone thickness and dental inclination on the lingual side of the bone, while the vestibular cortex did not depend on this angle.

In the present study, the angulation of the cortical plate was determined to evaluate the inclination of the vestibular alveolar surface and the mandibular body. This angle is diminished when teeth are inclined and/or when the anterior dimension of the mandible is increased due to prominent genial tuberosity. The presence of this tuberosity is the reason why angulation of the cortex assessed around canines is significantly higher than in incisors. Therefore, this angle cannot be used to evaluate inclination or tilting of the mandibular anterior teeth, but may be employed in implant planning. When the most lingually positioned implant is desired, there is a risk of the vestibular cortex bone being iatrogenically perforated by the implant apex and this risk is higher in the case of immediate implant placements when the longest possible implants are chosen in order to achieve primary stability. The risk also increases when the PQR angle is higher [1–3, 31]. In our own study, we found a correlation between higher PQR angle values and greater spongious bone thickness around the incisors and canines, while it did not influence cortical bone on both sides of the jaw.

Tooth rotation in relation to the midline increases for each successive tooth in the dental arch. The values of this angle are negative when teeth are rotated towards the midline and often differed from the mean value in our own material due to frequent dental crowding in the anterior mandible. However, our statistical analysis did not show any correlation between tooth rotation and bone thickness.

One of the limitations of the present study concerns the specially designed i-CAT Vision software used, which did not provide all the functionalities required to carry out planned measurements. Therefore, the images had to be exported to other software, which may limit the applicability of this method in everyday practice and increase potential measurement errors. Our evaluation was a one-time retrospective, and it would be advantageous to observe the dynamics of changes in bone volume in the anterior mandible during orthodontic treatment in terms of the location of teeth and the potential risk to periodontal status. We examined generally healthy patients. The results would probably have been different in patients with systemic diseases or in chronic drug use, which has an impact on the bone, e.g. hormone therapy.

## Conclusions

If CBCT is present during orthodontic treatment (or is taken for other reasons by the patient), it can be used to assess the presence of bone. The latter is surely needed in adults, and the effect of orthodontics should be further investigated on larger samples and prospectively during treatment.

## References

[1] Funato A, Salama MA, Ishikawa T, Garber DA, Salama H (2007). Timing, positioning, and sequential staging in esthetic implant therapy: a four-dimensional perspective. Int J Periodontics Restorative Dent.

[2] Jivraj S, Chee W (2006). Treatment planning of implants in the aesthetic zone. Br Dent J.

[3] Teughels W, Merheb J, Quirynen M (2009). Critical horizontal dimensions of interproximal and buccal bone around implants for optimal aesthetic outcomes: a systematic review. Clin Oral Implants Res.

[4] Ghassemian M, Nowzari H, Lajolo C, Verdugo F, Pirronti T, D’Addona A (2012). The thickness of facial alveolar bone overlying healthy maxillary anterior teeth. J Periodontol.

[5] Rossell J, Puigdollers A, Girabent - Farrés M (2015). A simple method for measuring thickness of gingiva and labial bone of mandibular incisors. Quintessence Int.

[6] Song JM, Lee JY, Kim YD (2015). CBCT morphologic analysis of edentulous posterior mandible for mandibular body bone graft. J Oral Implantol.

[7] Joss-Vassalli I, Grebenstein C, Topouzelis N, Sculean A, Katsaros C (2010). Orthodontic therapy and gingival recession: a systematic review. Orthodontics Craniofac Res.

[8] Seixas MR, Costa-Pinto RA, Araújo TMD (2012). Gingival esthetics: an orthodontic and periodontal approach. Dent Press J Orthodontics.

[9] Leymarie S (2012). Pre-orthodontic mucogingival surgery: an esthetical case report. J Dentofac Anom Orthodontics.

[10] Alhulaimi HA, Awartani FA (2013). Periodontium biotype modification prior to an orthodontic therapy: case report. King Saud Univ J Den Sci.

[11] Quereshy FA, Savell TA, Palomo JM (2008). Applications of cone-beam computed tomography in the practice of oral and maxillofacial surgery. J Oral Maxillofac Surg.

[12] Molen AD (2010). Considerations in the use of cone-beam computed tomography for buccal bone measurements. Am J Orthod Dentofac Orthop.

[13] Marmulla R, Wörtche R, Mühling J, Hassfeld S (2005). Geometric accuracy of the NewTom 9000 Cone-beam CT. Dentomaxillofac Radiol.

[14] Ferrare N, Leite AF, Caracas HCPM, de Azevedo RB, de Melo NS, Souza d, PT Fu (2013). Cone-beam computed tomography and microtomography for alveolar bone measurements. Surg Radiol Anat.

[15] Romero-Delmastro A, Kadioglu O, Currier GF, Cook T (2014). Digital tooth-based superimposition method for assessment of alveolar bone levels on cone-beam computed tomography images. Am J Orthod Dentofac Orthop.

[16] Leung CC, Palomo L, Griffith R, Hans MG (2010). Accuracy and reliability of cone-beam computed tomography for measuring alveolar bone height and detecting bony dehiscences and fenestrations. Am J Orthod Dentofac Orthop.

[17] Garib DG, Yatabe MS, Silva Filho OGD, Ozawa TO (2010). Alveolar bone morphology under the perspective of the computed tomography: defining the biological limits of tooth movement. Dent Press J Orthodontics.

[18] Kobayashi K, Shimoda S, Nakagawa Y, Yamamoto A (2003). Accuracy in measurement of distance using limited cone-beam computerized tomography. The. Int J Oral Maxillofac Implants.

[19] Mischkowski RA, Pulsfort R, Ritter L, Neugebauer J, Brochhagen HG, Keeve E, Zöller JE (2007). Geometric accuracy of a newly developed cone-beam device for maxillofacial imaging. Oral Surg Oral Med Oral Pathol Oral Radiol Endod.

[20] Timock AM, Cook V, McDonald T, Leo MC, Crowe J, Benninger BL, Covell DA (2011). Accuracy and reliability of buccal bone height and thickness measurements from cone-beam computed tomography imaging. Am J Orthod Dentofac Orthop.

[21] Zekry A, Wang R, Chau A, Lang NP (2014). Facial alveolar bone wall width - a cone-beam computed tomography study in Asians. Clin Oral Implants Res.

[22] Baysal A, Ucar FI, Buyuk SK, Ozer T, Uysal T (2013). Alveolar bone thickness and lower incisor position in skeletal Class I and Class II malocclusions assessed with cone-beam computed tomography. The. Korean J Orthodontics.

[23] Swasty D, Lee JS, Huang JC, Maki K, Gansky SA, Hatcher D, Miller AJ (2009). Anthropometric analysis of the human mandibular cortical bone as assessed by cone-beam computed tomography. J Oral Maxillofac Surg.

[24] Lee SL, Kim HJ, Son MK, Chung CH (2010). Anthropometric analysis of maxillary anterior buccal bone of Korean adults using cone-beam CT. J Adv Prosthodontics.

[25] Januário AL, Duarte WR, Barriviera M, Mesti JC, Araújo MG, Lindhe J (2011). Dimension of the facial bone wall in the anterior maxilla: a cone-beam computed tomography study. Clin Oral Implants Res.

[26] El Nahass HN, Naiem S (2015). Analysis of the dimensions of the labial bone wall in the anterior maxilla: a cone-beam computed tomography study. Clin Oral Implants Res.

[27] Wang HM, Shen JW, MF Y, Chen XY, Jiang QH, He FM (2014). Analysis of facial bone wall dimensions and sagittal root position in the maxillary esthetic zone: a retrospective study using cone-beam computed tomography. The. Int J Oral Maxillofac Implants.

[28] Braut V, Bornstein MM, Belser U, Buser D (2011). Thickness of the anterior maxillary facial bone wall—a retrospective radiographic study using cone-beam computed tomography. Int J Periodontics Restor Dent.

[29] Vera C, De Kok IJ, Reinhold D, Limpiphipatanakorn P, Yap AK, Tyndall D, Cooper LF (2011). Evaluation of buccal alveolar bone dimension of maxillary anterior and premolar teeth: a cone-beam computed tomography investigation. The. Int J Oral Maxillofac Implants.

[30] Nowzari H, Molayem S, Chiu CHK, Rich SK (2012). Cone-beam computed tomographic measurement of maxillary central incisors to determine prevalence of facial alveolar bone width≥ 2 mm. Clin Implant Dent Relat Res.

[31] Huynh-Ba G, Pjetursson BE, Sanz M, Cecchinato D, Ferrus J, Lindhe J, Lang NP (2010). Analysis of the socket bone wall dimensions in the upper maxilla in relation to immediate implant placement. Clin Oral Implants Res.

[32] Gracco A, Luca L, Bongiorno MC, Siciliani G (2010). Computed tomography evaluation of mandibular incisor bony support in untreated patients. Am J Orthod Dentofac Orthop.

[33] Ozdemir F, Tozlu M, Germec-Cakan D (2013). Cortical bone thickness of the alveolar process measured with cone-beam computed tomography in patients with different facial types. Am J Orthod Dentofac Orthop.

[34] Jonasson G, Kiliaridis S, Gunnarsson R (1999). Cervical thickness of the mandibular alveolar process and skeletal bone mineral density. Acta Odontol.

[35] Jonasson G, Kiliaridis S (2005). Changes in the bucco-lingual thickness of the mandibular alveolar process and skeletal bone mineral density in dentate women: a 5-yr prospective study. Eur J Oral Sci.

[36] Swasty D, Lee J, Huang JC, Maki K, Gansky SA, Hatcher D, Miller AJ (2011). Cross-sectional human mandibular morphology as assessed in vivo by cone-beam computed tomography in patients with different vertical facial dimensions. Am J Orthod Dentofac Orthop.

[37] Horner KA, Behrents RG, Kim KB, Buschang PH (2012). Cortical bone and ridge thickness of hyperdivergent and hypodivergent adults. Am J Orthod Dentofac Orthop.

[38] Schwarz AM (1961). Roentgenostatics: a practical evaluation of the x-ray headplate. Am J Orthod.

[39] Steiner CC (1960). The use of cephalometric as an aid to planning and assessing orthodontic treatment. Am J Orthod Dentofac Orthop.

[40] Wang RY, Han M, Liu H, Wang CL, Xian HH, Zhang L, Liu DX (2012). Establishment of reference mandibular plane for anterior alveolar morphology evaluation using cone-beam computed tomography. J Zhejiang Univ Sci B.

[41] Adams GL, Gansky SA, Miller AJ, WEJr H, Hatcher DC (2004). Comparison between traditional 2 - dimensional cephalometry and a 3-dimensional approach on human dry skulls. Am J Orthod Dentofac Orthop.

